# Supramolecular Chiral Discrimination of D-Phenylalanine Amino Acid Based on a Perylene Bisimide Derivative

**DOI:** 10.3389/fbioe.2020.00160

**Published:** 2020-03-04

**Authors:** Simona Bettini, Zois Syrgiannis, Michela Ottolini, Valentina Bonfrate, Gabriele Giancane, Ludovico Valli, Maurizio Prato

**Affiliations:** ^1^Department of Innovation Engineering, Campus University Ecotekne, University of Salento, Lecce, Italy; ^2^Consorzio Interuniversitario Nazionale per la Scienza e Tecnologia dei Materiali, Florence, Italy; ^3^Simpson Querrey Institute, Northwestern University, Chicago, IL, United States; ^4^Center of Excellence for Nanostructured Materials (CENMAT) and INSTM, Unit of Trieste, Department of Chemical and Pharmaceutical Sciences, University of Trieste, Trieste, Italy; ^5^Department of Biological and Environmental Sciences and Technologies (DiSTeBA), University of Salento, Lecce, Italy; ^6^Department of Cultural Heritage, University of Salento, Lecce, Italy; ^7^Ikerbasque, Basque Foundation for Science, Bilbao, Spain; ^8^Carbon Nanobiotechnology Laboratory, CIC biomaGUNE, Donostia-San Sebastian, Spain

**Keywords:** chiral discrimination, perylene bisimide, fluorescence spectroscopy, D-phenylalanine, supramolecular interaction

## Abstract

The interaction between homochiral substituted perylene bisimide (PBI) molecule and the D enantiomer of phenylalanine amino acid was monitored. Spectroscopic transitions of PBI derivative in aqueous solution in the visible range were used to evaluate the presence of D-phenylalanine. UV-visible, fluorescence, FT-IR, and AFM characterizations showed that D-phenylalanine induces significant variations in the chiral perylene derivative aggregation state and the mechanism is enantioselective as a consequence of the 3D analyte structure. The interaction mechanism was further investigated in presence of interfering amino acid (D-serine and D-histidine) confirming that both chemical structure and its 3D structure play a crucial role for the amino acid discrimination. A D-phenylalanine fluorescence sensor based on perylene was proposed. A limit of detection (LOD) of 64.2 ± 0.38 nM was calculated in the range 10^–7^–10^–5^ M and of 1.53 ± 0.89 μM was obtained in the range 10^–5^ and 10^–3^ M.

## Introduction

In the first half of the XIX century Louis Pasteur discovered, during his studies about the optical characteristics of a well-known chemical compound largely investigated in oenology, i.e., the tartaric acid, the chirality. Pasteur, most known for his important researches on the veterinary medicine, human health, and agriculture, detailed the molecular chirality of the tartaric acid by means of crystallographic and optical activity characterizations starting a new direction in chemistry (Pasteur, 1853; Gal, 2017). Starting from Pasteur’s observations, it was more and more clear that the 3-Dimensional configuration of organic molecules remarkably affects their chemical activity (Xiao et al., 2016). For example, the 3D shape of protein arrangement is crucial for the enzyme assembly (Banik and Nandi, 2012). There is a plethora of chiral molecules in nature, with the most characteristic examples being sugars and amino acids (Kabsch and Sander, 1983; Mason, 1984; Klemm et al., 2005). Amino acids exist in two different enantiomers, L and D form, even though is not clear the reason why the Nature almost exclusively use the L enantiomer. Another example, the nucleic acids are built by base-pairs with the same chirality (Young et al., 2019). These natural examples worked as inspiration for the synthesis of several molecules, which are used for pharmaceutical applications (Nguyen et al., 2006), and based on well-defined chiral forms. So it may be the case that an enantiomer produces an effect on a living organism and the opposite chiral form will produce no effect or even consequences harmful to health (Vargesson, 2015). Once more, the presence of D-amino acids in natural products can represent an alert for bacterial contamination (Trojanowicz and Kaniewsk, 2011; Zor, 2018).

In this context, the possibility for efficient discrimination of a chiral form from another one appears a very appealing goal and several approaches and transduction methods for such recognition have been proposed (Zor et al., 2019). Chiral sensors are mainly based on the host-guest interaction between an active molecule and the chiral analyte driven by hydrogen bonds, π–π interactions etc. (Huang et al., 2016; Wang et al., 2019). For the realization of the active layer both natural and synthetic materials are used (Torsi et al., 2008; Arnaboldi et al., 2018). The possibility to tailor the structures in order to promote highly selective chiral recognition and the relative simple procedure to obtain chiral substrates suggest the artificial materials as a preferential approach to obtain chiral sensors (Tiwari and Prasad, 2015; Arnaboldi et al., 2018). As transduction methods, electrochemical, and electronic approaches have been successfully proposed (Fireman-Shoresh et al., 2005; Ariga et al., 2010; Duan et al., 2015; Stefanelli et al., 2019; Zhang et al., 2019; Zor et al., 2019). Furthermore, optical and spectroscopic methods are successfully used for realizing chiral sensors. Optical methodologies, by monitoring the changes induced in the UV-visible absorption spectrum when an enantiomer of a chiral specie is detected, appear particularly fascinating since the transduction method is very simple and the results analysis is in the most cases intuitive (Sortino et al., 2003; Chen et al., 2015; Buccolieri et al., 2018). The approach that was used in the present work can be considered as the initial point to use chiral perylene bisimide (PBI) based systems for the detection of specific chiral form. The supramolecular chiral discrimination of a single enantiomer over the other is reported even for other systems (Cooper et al., 2017; Gangopadhyay et al., 2017; Noguchi et al., 2017). We present here the symmetric PBI with D-phenylalanine in the amide position which acts as a chiral sensor only for the D form of the phenylalanine. According to our knowledge this type of recognition can pave the road in the direction of chiral discrimination (Avinash and Govindaraju, 2012; Sato et al., 2019; Shang et al., 2019).

L-phenylalanine is an essential amino acid and it is used in the human metabolism to build up proteins and to obtain tyrosine, another amino acid that is needed to make proteins and brain chemicals (Cansev and Wurtman, 2007; Fernstrom and Fernstrom, 2007). On the contrary, D-phenylalanine is synthetized in laboratory and its role in the living organisms is not currently well-understood. D-phenylalanine has been proposed to treat chronic pain (Russell and McCarty, 2000) even though this effect is not completely accepted from the academic community. Anyway, D-amino acids are used as intermediates for the synthesis of β-lactam antibiotics (Breuer et al., 2004; Hanson et al., 2008). In particular, D-phenylalanine is used to obtain the nateglinide, a compound belonging to the group of the meglitinides, used to treat type 2 diabetes (Tentolouris et al., 2008). Furthermore, D-phenylalanine is used as analgesic and antistress agent (Ruilope, 2003; Gao et al., 2013). Here we report the possibility to readily discriminate D-phenylalanine in water solution by means of UV-visible and fluorescence spectroscopy and, above all, the reported results aim for demonstrating the possibility to discriminate the amino acids enantiomer by using chiral substituted chromophores such as PBI derivative.

## Experimental Section

### Materials

**FIGURE 1:**
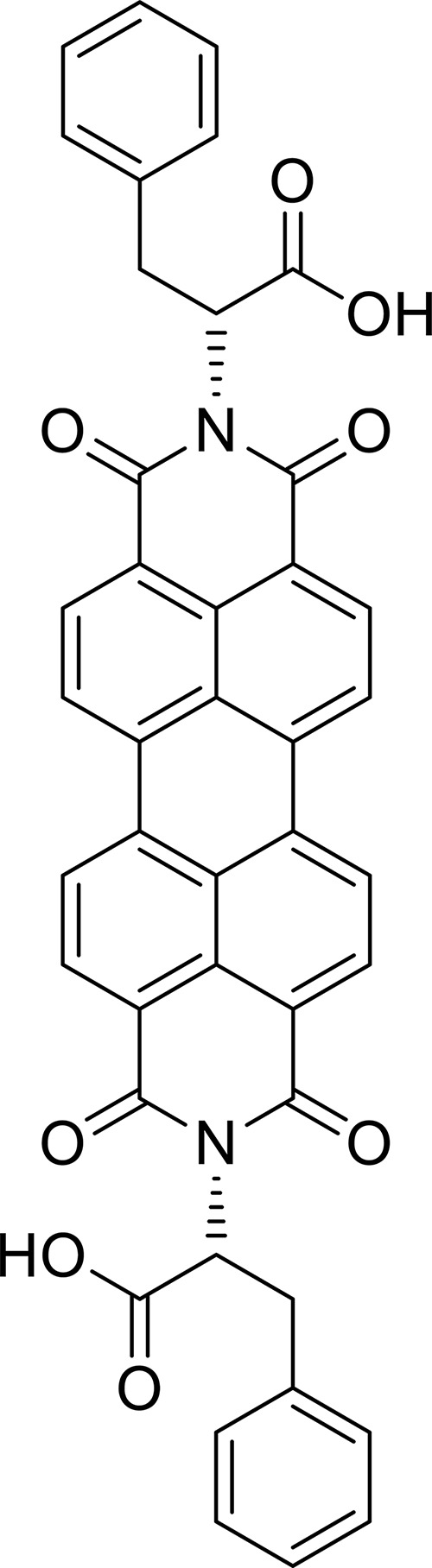
Perylene bisimide (PBI) derivative chemical structure.

The chemical structure of the PBI derivative (D-PBI) is reported in Figure 1. It was obtained according to the procedure reported in the literature (Gershberg et al., 2015).

Milli-Q grade water was used to dissolve the PBI derivative and all the organic compounds (D-phenylalanine, L-phenylalanine, D-serine, D-histidine), used in this contribution, were purchased from Sigma-Aldrich and used as received.

### Methods

UV-visible absorption spectroscopy characterization was performed by means of a Varian Cary 5000 and allowed to study the aggregation state of D-PBI in aqueous solution at different concentrations and, after fixing the PBI concentration, in presence of the different tested analytes. The fluorescence emission properties of D-PBI were investigated as well. Aqueous solutions were prepared and analyzed upon excitation at 500 nm by using a Fluorolog Jobin Yvon (Horiba) instrument. Spectra were recorded between 510 and 700 nm in order to detect the two emission peaks located at about 546 and 590 nm. The fluorescence enhancement at 546 nm due to the interaction with D-phenylalanine was, consequently, monitored and the semilogarithmic dependence of the fluorescence enhancement as function of analyte concentration was obtained.

Fourier transform infrared (FT-IR) Spectrum One (PerkinElmer) in attenuated total reflection (ATR) mode was used to characterize the PBI derivative and the interaction with the chiral amino acids. Spectra of D-PBI and of D-PBI mixed with D-phenylalanine and L-phenylalanine were obtained by depositing a drop of the aqueous solution directly on the ATR prism. After solvent evaporation, spectra were recorded with 4 cm^–1^ resolution and an average scan number of 32.

Atomic Force Microscope (AFM) characterization was performed on a NanoSurf SPM S200 microscope. The films were prepared by drop casting on silicon slides. This morphological characterization allowed to investigate the elongated aggregates of D-PBI alone or in presence of analytes.

PBI derivative aggregates dimension in solution was evaluated by means of nanoPartica SZ-100 (Horiba). The dimension change upon interaction with the analyte was evaluated as well.

## Results and Discussion

### Spectroscopic Characterization of D-PBI Aqueous Solution

**FIGURE 2:**
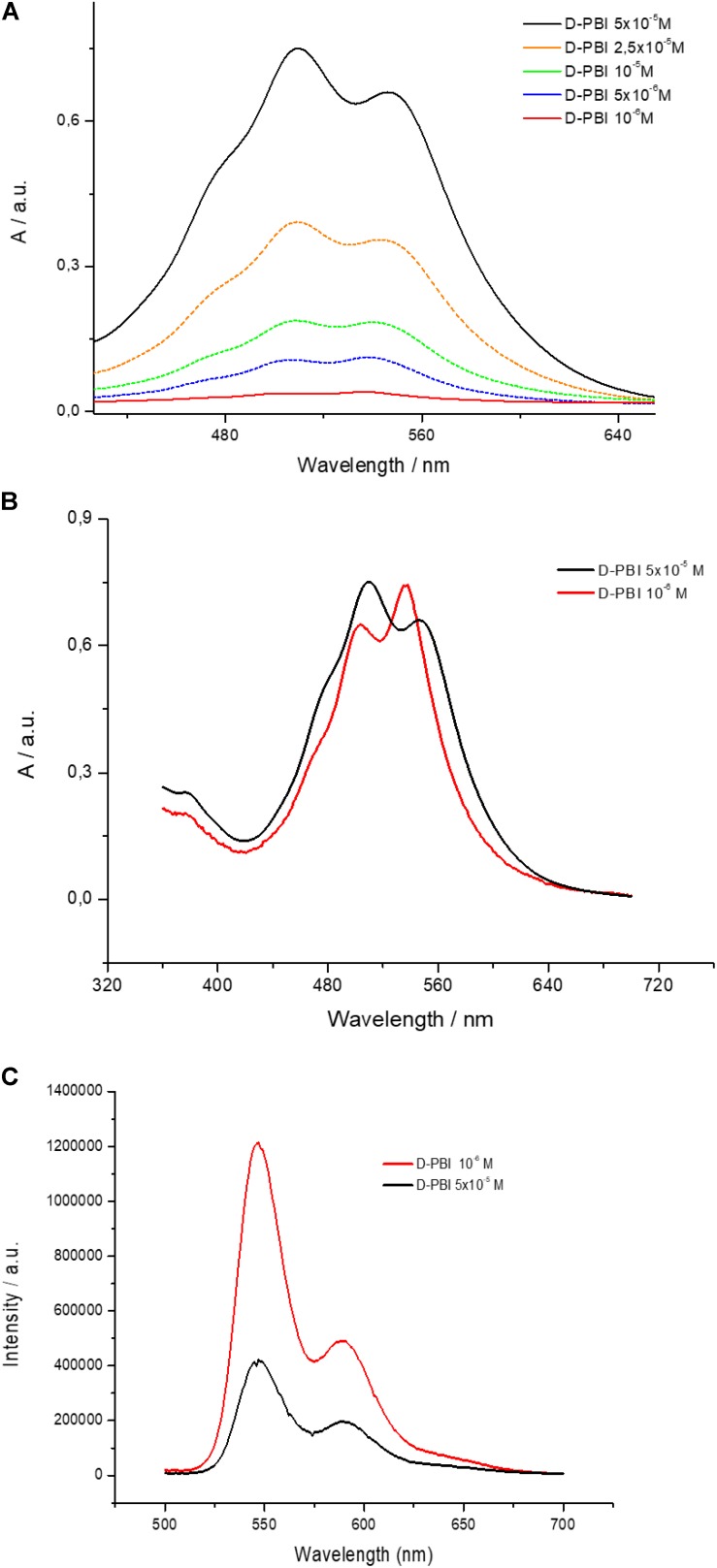
**(A)** UV-visible spectra of D-PBI aqueous solutions at different concentrations in the 420–650 nm range; **(B)** A comparison of UV-visible spectrum of an aqueous solution of D-PBI 10^–6^ M (multiplied by 30 times) and of an aqueous solution of D-PBI 5 × 10^–5^ M; **(C)** Emission spectra (λ_*exc*_ = 500 nm) of an aqueous solution of D-PBI 10^–6^ M and of an aqueous solution of D-PBI 5 × 10^–5^ M.

PBI derivative aqueous solutions (in Milli-Q grade water) at different concentrations, ranging from 10^–6^ M to 5 × 10^–5^ M, were prepared and analyzed by means of UV-visible absorption spectroscopy. The obtained spectra, in the 420–650 nm range, are reported in Figure 2A. D-PBI concentrations below 10^–6^ M have not been investigated, by means of this approach, since at 10^–6^ M the absorbance becomes lower than 0.1 (red line, Figure 2A). Instead, the highest concentration has been fixed at 5 × 10^–5^ M in order to work in the Lambert-Beer linear range (Gershberg et al., 2015). Generically, PBI derivatives are characterized by two main absorption peaks located at about 500 nm and 537 nm, corresponding to the 0 → 1 and 0 → 0 transitions, respectively (Chen et al., 2012), which can be monitored in terms of wavelength peak position and intensity ratio (*A_0→0_*/*A_0→1_*) to obtain information about the aggregation state of the PBI molecules (Burian et al., 2017). D-PBI spectrum, indeed, has been demonstrated to be dominated by the presence of these two signals, which are located, for the 5 × 10^–5^ M solution (black line, Figure 2A) at 510 nm and 546 nm. The recorded red shift of about 10 nm for both signals could be imputable to the presence of D-PBI aggregates in solution upon π–π stacking events (Chen et al., 2012, 2018). Moreover, by calculating the *A_0→0_*/*A_0→1_* ratio value (0.9) at this concentration, the presence of both π–π aggregates, in particular H aggregates, and monomeric form could be hypothesized. In fact, it is reported that for *A_0→0_*/*A_0→1_* values below 0.7, PBI molecules are organized as H aggregates in aqueous solutions, whilst for *A_0→0_*/*A_0→1_* values of about 1.6 the monomeric form is dominant (Chen et al., 2012). A value of 0.9 might correspond to a mixed situation, in which both H aggregates and monomeric form are present in solution, but with an aggregates excess. By analyzing the other spectra (Figure 2A), the simultaneous presence of aggregates and monomers was confirmed for all the investigated cases, even if the *A_0→0_*/*A_0→1_* value increases when D-PBI concentration decreases down to a value of 1.1 in the most diluted investigated solution, suggesting that upon dilution the aggregates/monomer equilibrium can be moved toward the monomeric form. The *A_0→0_*/*A_0→1_* values were plotted in function of D-PBI molar concentration and are reported in Supplementary Figure S1.

This rationale was confirmed by the recorded blue shift when D-PBI solution was gradually diluted, suggesting the presence of monomers in solution (Langhals et al., 1991, 1998; Langhals, 1995; Chen et al., 2012). Figure 2B reports a comparison of the spectra obtained for D-PBI at 5 × 10^–5^ M (black line) and at 10^–6^ M (red line). The last one was multiplied by 30 to make easier the comparison. The red spectrum is indeed characterized by the 0 → 0 transition band located at 537 nm and the 0 → 1 at 500 nm. Not only, but an inversion of the two contributions intensity appears evident.

Steady state fluorescence spectroscopy investigations have been carried out and are in good agreement with the obtained results. The performed emission spectra upon illumination at 500 nm are reported in Figure 2C, for D-PBI at 5 × 10^–5^ M (black line) and for D-PBI at 10^–6^ M (red line). Two signals located at 550 nm and 590 nm can be detected. Even though the red spectrum arises from a 50 times more diluted solution, it is about three times more intense than the black spectrum. This phenomenon, again, assessed the existence of a monomer/aggregates equilibrium (Chen et al., 2012), and in particular is due to the presence of a higher concentration of the monomer in the 10^–6^ M solution, according to the *A_0→0_*/*A_0→1_* calculated values (Supplementary Figure S1). PBI monomer, generically, is characterized by a higher emission fluorescence compared to both H aggregates and J aggregates (Chen et al., 2012).

In Supplementary Figure S2 the FT-IR spectrum obtained by depositing a cast film of D-PBI aqueous solution directly on the ATR prism in the 4000–1000 cm^–1^ frequency range has been depicted. The spectrum is characterized by the typical vibrations of PBI derivatives (Semeraro et al., 2019) and some signals due to phenylalanine moieties. The asymmetric signal located at about 3400–3000 cm^–1^ is most probably due to phenylalanine substituents νO-H and νN-H modes, whilst the two signals between 2950 and 2800 cm^–1^ arise from aromatic C-H and CH_2_ stretching modes of both D-PBI and phenylalanine substituents. The two contributes located at 1696 and 1655 cm^–1^ are ascribable to asymmetric and symmetric stretching of the imide C = O of D-PBI and the signals located at 1592 and 1574 cm^–1^ are due to PBI and phenylalanine νC = C (aromatic) and to the amino acid N-H scissoring. The vibrational bands in the 1450–1330 cm^–1^ frequency range and in the range 1260–1150 cm^–1^ are ascribable to –CH bending modes and the C-N stretching modes, respectively (Bettini et al., 2019).

### Disaggregation Process of D-PBI Aqueous Solution in Presence of D-Phenylalanine

**FIGURE 3:**
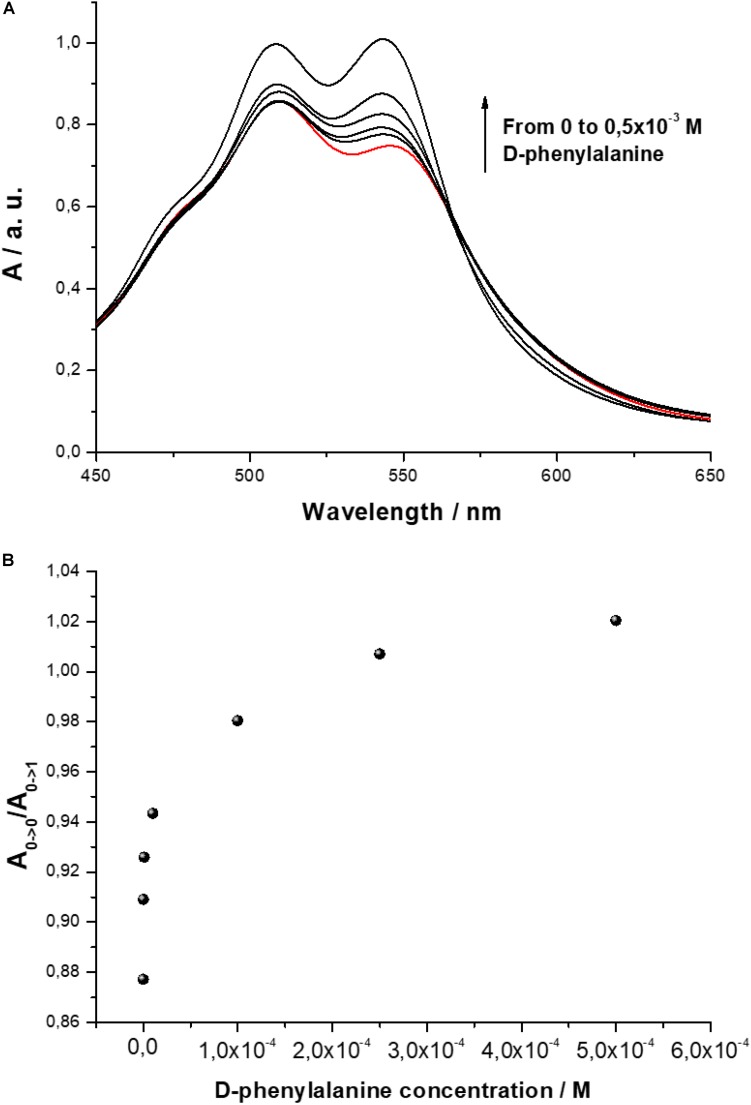
**(A)** UV-visible spectrum of D-PBI aqueous solution (5 × 10^–5^ M) and in presence of increasing concentration of D-phenylalanine (10^–7^ M, 10^–6^ M, 10^–5^ M, 10^–4^ M, 0.5 × 10^–3^ M); **(B)** Ratio *A_0→0_*/*A_0→1_* variation as a function of different concentrations of D-phenylalanine.

The interaction among D-PBI and D-phenylalanine molecules was monitored by means UV-visible using a 5 × 10^–5^ M water solution of the perylene derivative. An increasing concentration of D-phenylalanine, starting from 10^–7^ M, was mixed with D-PBI solution and the spectral variations were monitored in the range 450–650 nm (Figure 3A).

The ratio between the bands located at 546 and 510 nm, that is related to the aggregation state of the PBI derivative, is influenced by the presence of D-phenylalanine. In Figure 3B the ratio *A_0→0_*/*A_0→1_* is plotted. When a small amount of D-phenylalanine is added, a relevant variation of the relative peak intensities is observed; increasing the analyte concentration, the effect of the D-phenylalanine on the aggregation state of the PBI derivatives appears almost asymptotic.

**FIGURE 4:**
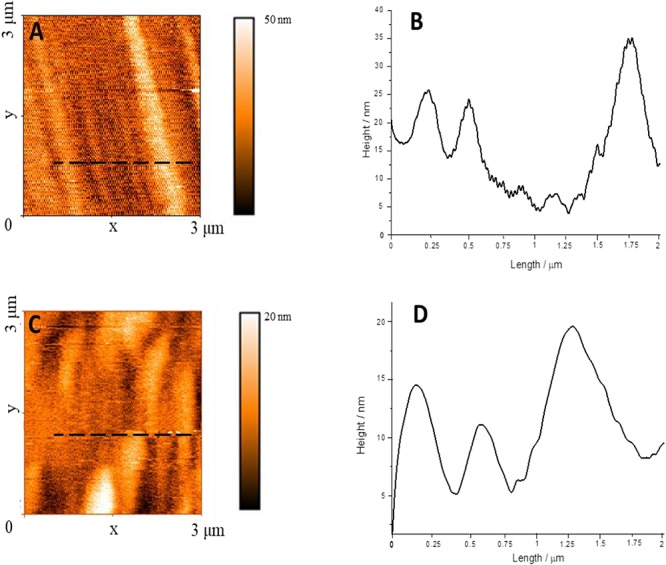
**(A)** AFM image of D-PBI on silicon substrate; **(B)** Line profile recorded along the black dotted line in box **(A)**; **(C)** AFM image of a cast film of D-PBI after the interaction with D-phenylalanine; **(D)** Line profile recorded along the black dotted line in box **(C)**.

According with the literature (Bettini et al., 2019), the aggregation state of PBI can be drastically influenced by the interaction with guest molecules with specific chemical features. In the present work, in order to confirm the spectroscopic evidences, i.e., that the D-phenylalanine influences the aggregation of D-PBI, a morphological study on cast films of the perylene derivative before and after the interaction with D-phenylalanine was carried out by means of atomic force microscopy (Figure 4).

The D-PBI aggregates appear as well-defined structures of about 250 nm width (Figure 4A and Supplementary Figures S3, S4), some μm long and up to 40 nm high (Walsh et al., 2016; Draper et al., 2019; Guo et al., 2019). When the D-PBI is casted after the interaction with D-phenylalanine the elongated structure appears shorter with larger diameter (20 nm higher than the pristine D-PBI). This suggests that the presence of D-phenylalanine influences the D-PBI aggregates, confirming the spectroscopic evidences. So, what we can propose is that D-phenylalanine molecules induce a disaggregation of D-PBI supramolecular structures by presumably reducing the π–π stacking. In fact, the D-PBI aggregates hydrodynamic size was evaluated in solution in presence or not of D-phenylalanine. The *Z*-average value obtained in the DLS measurements confirmed the influence of the D form of the amino acid on the size of the D-PBI aggregates. A 5 × 10^–5^ M aqueous solution of D-PBI showed a hydrodynamic diameter of *ca* 254 nm, in very good agreement with AFM investigations. In presence of D form of analyte this value increases up to *ca* 690 nm, being in accordance with AFM measurements.

It is interesting to observe that the aggregation state of D-PBI is not influenced when L-phenylalanine is used as the analyte molecule (Supplementary Figure S5). Based on these evidences we can conclude that the interaction is strongly enantioselective. It can be supposed that the electron-donating amine group of D form of phenylalanine interacts with the electron-withdrawing imide group of D-PBI (Bettini et al., 2019) and the aromatic moiety of the amino acid interacts with the aromatic ring of the phenylalanine substituent (Giancane et al., 2013; Bettini et al., 2015). The formation of the host-guest complex, allowing the insertion of D-phenylalanine moieties within the aggregates, reduces the stacking of the PBI derivative as highlighted by AFM and visible spectroscopy.

On the contrary, when the L form of phenylalanine is mixed with D-PBI, the aromatic overlapping between the aromatic ring of the amino acid and the substituent on the perylene does not take place, probably due to the different 3D conformation of the enantiomer. Moving one step further we used a racemic solution of L and D phenylalanine which is mixed with D-PBI solution. The band ratio *A_0→0_*/*A_0→1_* is changed in half value, according to the D enantiomer concentration in the racemic mixture (Supplementary Figure S5). Consequently, both forms of the amino acid are able to interact with the active molecules, but only the D form is able to disaggregate the supramolecularly arranged D-PBI structures. We can presume that both D and L phenylalanine amine group interacts with the imide C=O of D-PBI, but the position of the aromatic ring, determined by the chirality, results available only for the D form. According to this rationale, FT-IR spectroscopy investigation (Supplementary Figure S6), carried out on a cast film of D-PBI (5 × 10^–5^ M), a cast film of D-PBI (5 × 10^–5^ M) mixed with D-phenylalanine (10^–6^ M), and a cast film of D-PBI (5 × 10^–5^ M) mixed with L-phenylalanine (10^–6^ M), showed that only the vibrational signals arising from the imide group of D-PBI in the range 1740–1560 cm^–1^ and 1300–1200 cm^–1^ change upon interaction with both enantiomers underlying the role played by this chemical group. Instead, the aromatic C=C signal (1590 cm^–1^) is dominated by the PBI core and we cannot distinguish the role played by the aromatic ring of the substituents.

According with the estimation of the activation energy in the de-assembling process of the aggregates as a consequence of the interaction with D-phenylalanine molecules (Schill et al., 2017; Stovbun et al., 2018; Zlenko et al., 2019a), the binding energy involved for the reported mechanism appears not strong enough (Park and Lee, 2006; Sriramulu and Valiyaveettil, 2016) for inducing a monomerization of D-PBI. The so-called “spontaneous resolution” can be proposed as a cooperative mechanism for justifying the chiral recognition of phenylalanine amino acid (Zlenko et al., 2019b). According to this fascinating theory, the separation of two enantiomers could be obtained as amplification of the initial asymmetry under the effect of some chemical compounds already present in the system (Stovbun et al., 2019). Furthermore, an additional effect could be represented by the spin polarization that was demonstrated to play an important role in the chiral biomolecular interactions (Kumar et al., 2017; Dalum and Hedegård, 2019; Naaman et al., 2019).

As a further confirmation of the role of the chemical structure of both chiral PBI and D-phenylalanine, we evaluated the effect of an amino acid without the aromatic group. In particular, D-serine was mixed with D-PBI at a concentration of 10^–4^ M in aqueous solution. As reported in Supplementary Figure S7, the effect of D-serine on the aggregation of D-PBI is almost negligible, confirming the crucial role played by the aromatic moiety of the analyte as well as its enantiomer form (Bettini et al., 2019). Again, the effect of D-histidine was evaluated. As expected, the aggregation state of D-PBI is influenced by D-histidine presence, even though the effect is reduced if compared with the *A_0→0_*/*A_0→1_* variation induced by D-phenylalanine (Supplementary Figure S8). It is a consequence of the presence of an aromatic heterocycle on histidine: it reduces the π–π interaction with the phenylalanine substituent on D-PBI, reducing the disaggregation of PBI derivative stacking.

**FIGURE 5:**
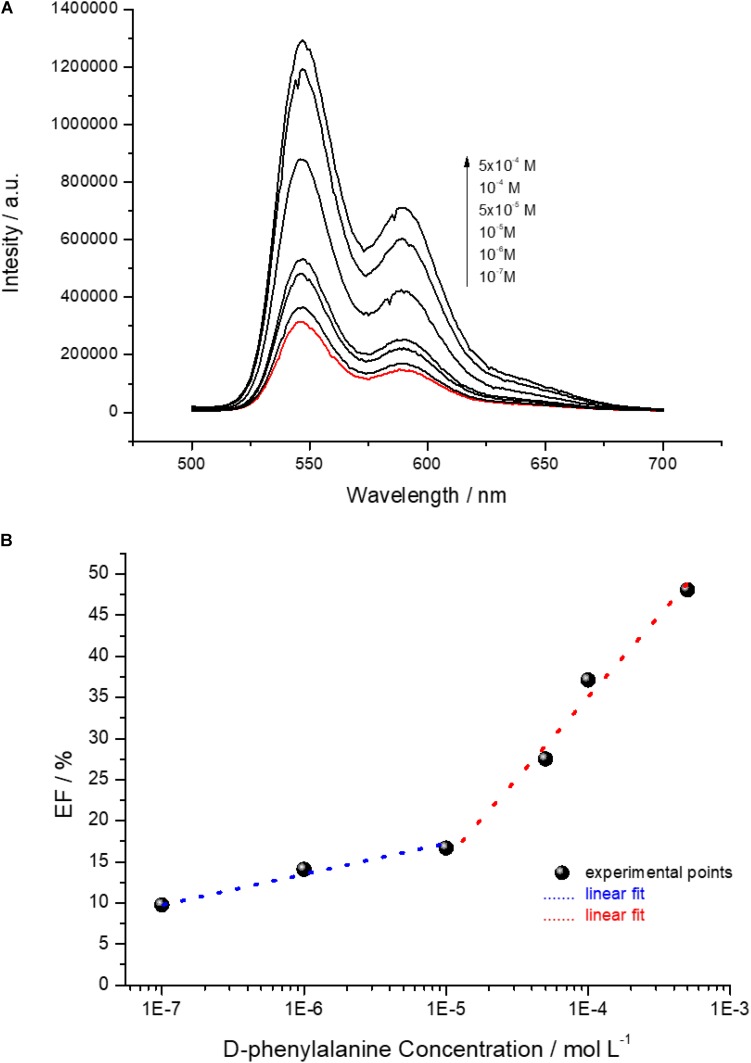
**(A)** Emission spectra (λ_*exc*_ = 500 nm) of an aqueous solution of D-PBI 5 × 10^–5^ M (red line) and of D-PBI 5 × 10^–5^ M in presence of different D-phenylalanine molar concentrations; **(B)** EF (%) of D-PBI emission intensity recorded at 546 nm depending on different D-phenylalanine molar concentration.

The D-phenylalanine capability to disassemble the D-PBI aggregates was, indeed, exploited to develop a preliminary detection system in solution by means of fluorescence spectroscopy, as proof of concept for chiral discrimination of phenylalanine enantiomers. The emission spectra (λ_*exc*_ = 500 nm) of D-PBI (5 × 10^–5^ M) in presence of different concentrations of the D form of the amino acid are reported in Figure 5A and clearly demonstrated that the enantiomer induces an enhancement of PBI derivative fluorescence, according to the proposed disaggregation mechanism of interaction. In fact, upon phenylalanine concentration increase, PBI derivative fluorescence intensity has been demonstrated to become more intense as well. This is in good agreement with the aforementioned experimental results (Figure 2C), the reduction of the PBI aggregation state is correlated to an enhancement of fluorescence emission. In presence of phenylalanine, indeed, the π–π stacking among D-PBI moieties is most probably reduced by the steric hindrance of the amino acid inserting within the aggregates, reducing the self-quenching events (Chal et al., 2018; Bettini et al., 2019). Then, the enhancement factor (EF) of D-PBI fluorescence (λ_*exc*_ = 500 nm) in presence of phenylalanine at different concentrations was calculated as

$$\mathrm{EF}=\frac{I_{i}-I_{0}}{I_{0}}\times 100$$

where *I*_0_ is referred to the fluorescence intensity at 590 nm of a solution of D-PBI 5 × 10^–5^ M and *I_i_* to the fluorescence intensity at 546 nm of a solution of D-PBI 5 × 10^–5^ M mixed with phenylalanine at various concentrations. Two different linear phenylalanine molar concentration ranges of response, in semilogarithmic scale, were found for the developed detection system (Figure 5B).

The first range was found between 10^–7^ M and 10^–5^ M with a correlation coefficient of about 0.98 and second in the 10^–5^–10^–3^ M range with a correlation coefficient of about 0.97. The limit of detection (LOD) was calculated for both the lower and higher concentration range (Vergara et al., 2018):

$$\mathrm{LOD}=\frac{\sigma \times F}{b}$$

where σ is the standard deviation (calculated by monitoring the emission intensity on a blank sample for 10 different samples), *F* a factor corresponding to 3.3 (Stephen et al., 2009; Bolton and Bon, 2010; Shrivastava and Gupta, 2011) and b the slope of the regression line. The calculated LOD for the lower concentration range of response is 64.2 ± 0.38 nM and for the higher concentration range is 1.53 ± 0.89 μM. Moreover, the D-PBI based system is characterized by two different analytical sensitivity values, calculated as the lowest phenylalanine detected concentration (mol L^–1^) divided by the correspondent EF value, corresponding to 80 nM/% in the lower concentration range and 60 μM/% in the higher one. The calculated LOD value, in particular 64.2 ± 0.38 nM, is a very promising result, considering that we worked in solution by just mixing the active molecules and the analyte and we already obtained a LOD of a few nanomol L^–1^. The analytical sensitivity of the system, indeed, especially in the 10^–7^ M–10^–5^ M phenylalanine range, is very encouraging for the further design, development and engineering of an optical sensing device for phenylalanine enantiomers discrimination. It is reasonable supposing that the use of more sensitive transduction method, such as Surface Plasmon Resonance or Surface Enhanced Raman Spectroscopy, could strongly improve the limit of the detected amount of D-phenylalanine by means of D-PBI.

## Conclusion

Homochiral substituted perylene with D-phenylalanine was used to selectively discriminate D enantiomer of the amino acid phenylalanine. It was demonstrated by means of spectroscopic techniques, and in particular studying the UV-visible spectrum, that the aggregation state of the D-PBI in water solution is strongly influenced by the presence of D-phenylalanine; on the contrary L-phenylalanine effect is negligible. The *A_0→0_*/*A_0→1_* ratio value can be used to evaluate the aggregation form of the PBI derivative. When D-PBI aqueous solution (5 × 10^–5^ M) cast film is characterized by means of AFM, well-defined elongated structure appears; when the PBI derivative is cast after the interaction with D-phenylalanine the supramolecular structures appear partially disaggregated as a consequence of the reduction of the π–π stacking.

The interaction between D-PBI and phenylalanine is strongly enantioselective. It can be supposed that the electron-donating amine group of D form of phenylalanine interacts with the electron-withdrawing imide group of D-PBI and the aromatic moiety of the amino acid interacts with the aromatic ring of the phenylalanine substituent. We can presume that both D and L phenylalanine amine group interacts with the imide C=O of D-PBI, but the position of the aromatic ring, determined by the chirality, results available only for the D form. The presence of the aromatic ring on the amino acid is necessary to modify the aggregation state of the D-PBI aggregates making the active molecule highly selective toward D-phenylalanine.

Furthermore, as well-known, the fluorescence emission is strongly influenced by self-quenching phenomena and, more in general, the aggregation form of a fluorophore can be crucial for the shape and intensity of the fluorescence emission. The D-PBI fluorescence enhancement was monitored in presence of different amounts of D-phenylalanine. A LOD of 64.2 ± 0.38 nM was calculated in the range 10^–7^–10^–5^ M and of 1.53 ± 0.89 μM was obtained for D-phenylalanine dissolved in ultrapure water in the range 10^–5^ and 10^–3^ M.

Concluding, the D-phenylalanine ability to influence the D-PBI aggregation was used to develop a preliminary detection system as proof of concept for chiral discrimination of phenylalanine enantiomers.

## Data Availability Statement

The datasets generated for this study are available on request to the corresponding author.

## Author Contributions

ZS synthetized PBI derivative under the supervision of MP and was actively involved in the results discussion about PBI aggregation state. MO and VB performed the UV–Vis and Fluorescence Emission spectroscopy measuraments under the supervision of GG. SB carried out the FT-IR, AFM, and DLS measurements under the supervision of LV and was actively involved in the results discussion about the host–guest interaction mechanism with GG, MO, and LV. All the authors contributed to arrange the manuscript.

## Conflict of Interest

The authors declare that the research was conducted in the absence of any commercial or financial relationships that could be construed as a potential conflict of interest.
