# Supplementary material for: Supramolecular Chiral Discrimination of D-Phenylalanine Amino Acid Based on a Perylene Bisimide Derivative
Source: Front Bioeng Biotechnol. 2020 Mar 4;8:160. doi: 10.3389/fbioe.2020.00160 (PMC7064719; doi:10.3389/fbioe.2020.00160)
Supplement: Supplementary file 1 [file Data_Sheet_1.pdf]

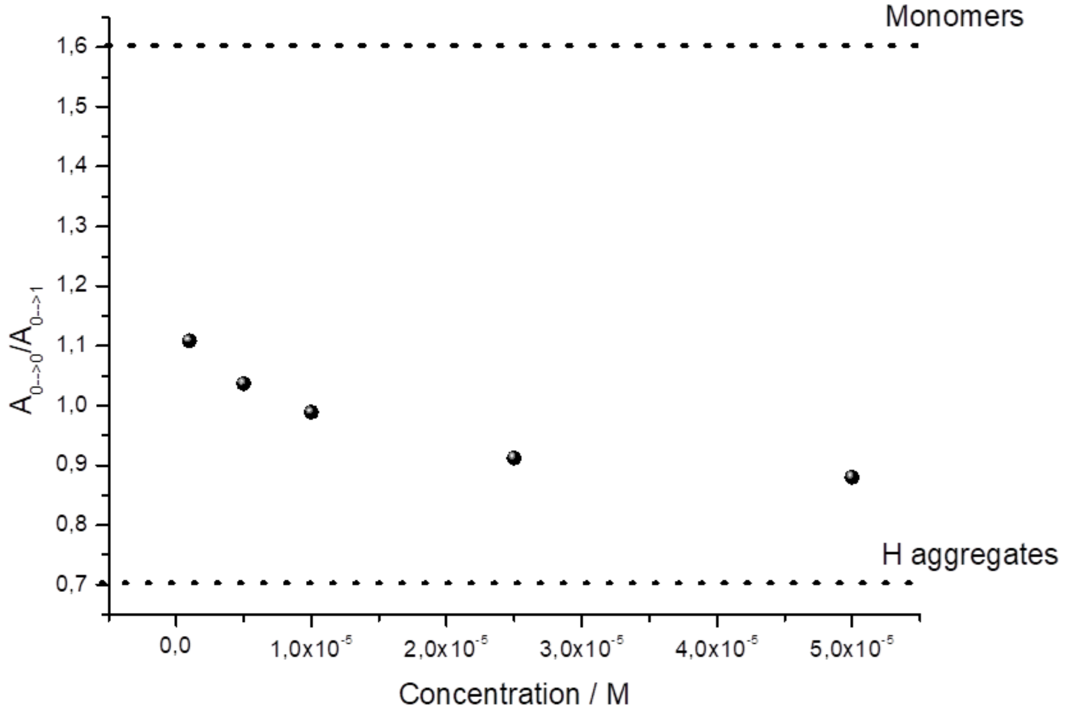

Figure S1: $A_{0\to0}/A_{0\to1}$ values calculated for D-PBI aqueous solutions at different concentrations; below 0.7 the solution should present just H aggregates, over 1.6 just monomeric forms.

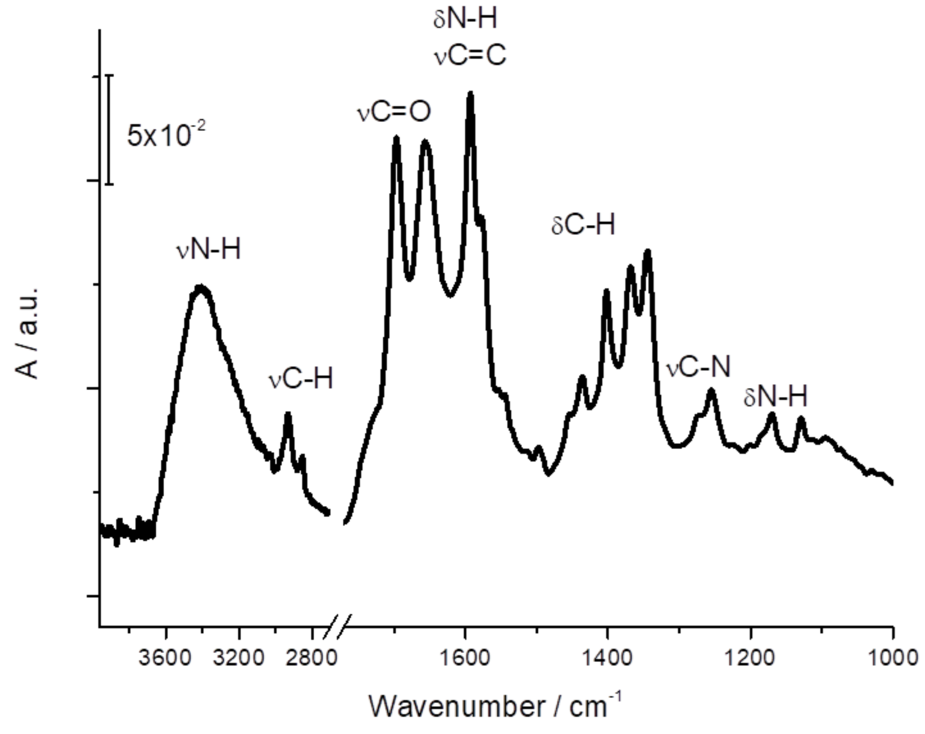

Figure S2: FT-IR spectrum of a cast film of D-PBI in the 4000-1000 cm^{-1} range.

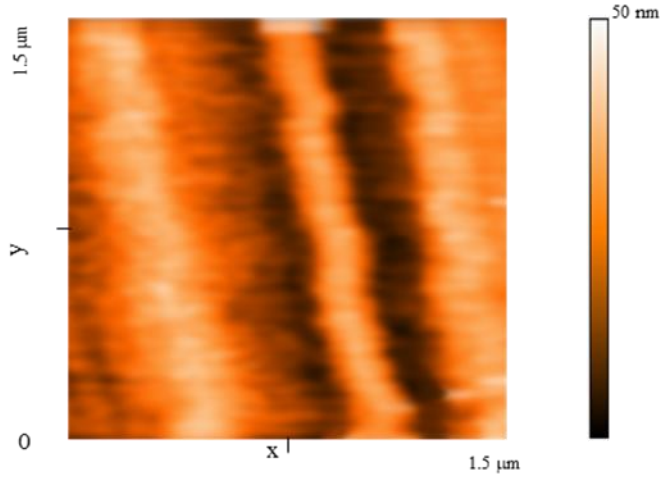

Figure S3: AFM image of elongated structures of D-PBI on silicon substrate.

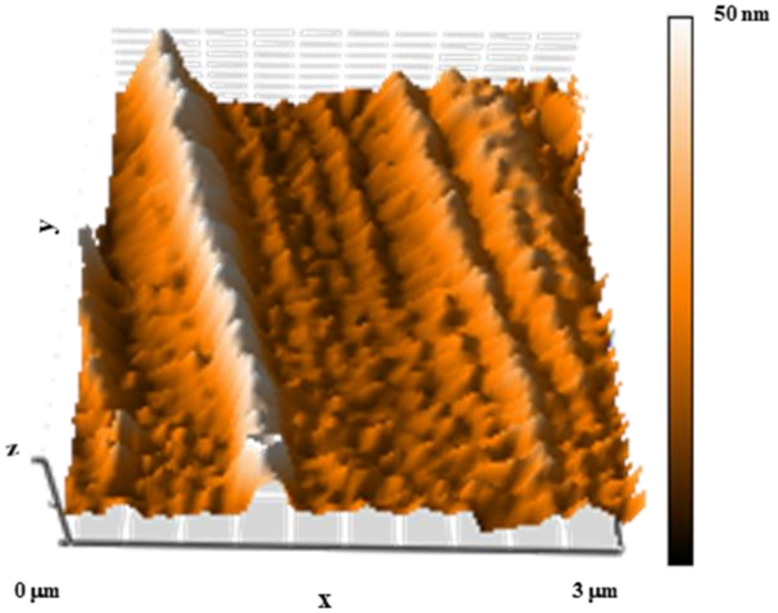

Figure S4: 3D AFM image of D-PBI on silicon substrate.

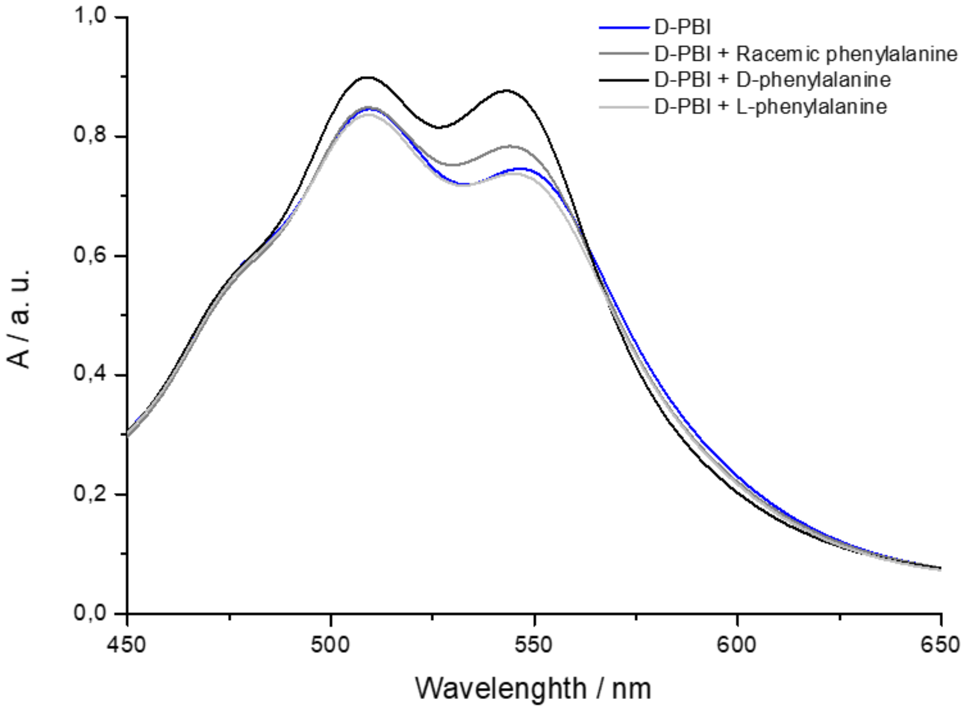

Figure S5: UV-visible spectrum of D-PBI aqueous solutions ($5x10^{-5}$ M) and comparison with D-PBI aqueous solutions ($5x10^{-5}$ M) mixed with racemic form of phenylalanine (10^{-4} M), D-phenylalanine (10^{-4} M) and L-phenylalanine (10^{-4} M).

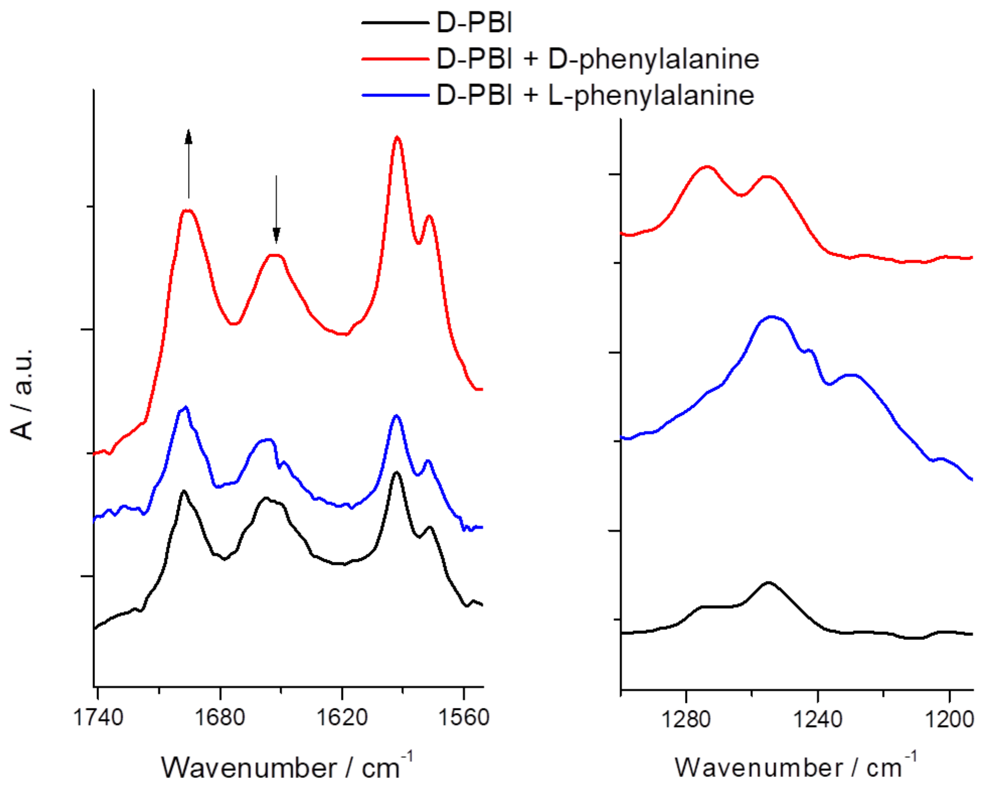

Figure S6: FT-IR spectrum of a cast film of D-PBI ($5x10^{-5}$ M), a cast film of D-PBI ($5x10^{-5}$ M) mixed with D-phenylalanine (10^{-6} M) and a cast film of D-PBI ($5x10^{-5}$ M) mixed with L-phenylalanine (10^{-6} M) in the range 1740-1560 cm^{-1} and 1300-1200 cm^{-1}.

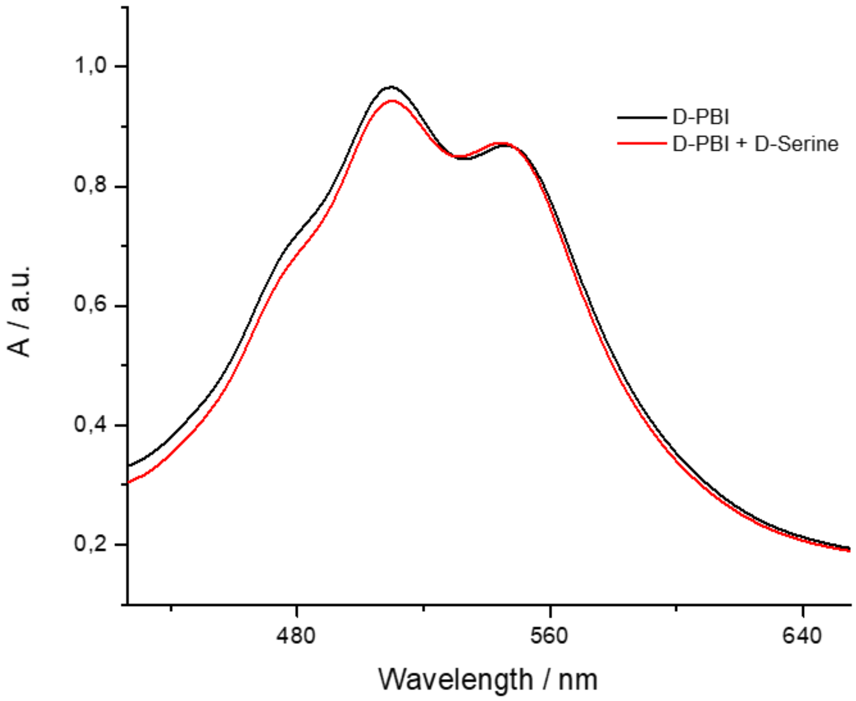

Figure S7: UV-visible spectra of D-PBI aqueous solutions ($5x10^{-5}$ M) and mixed with D-serine (10^{-4} M).

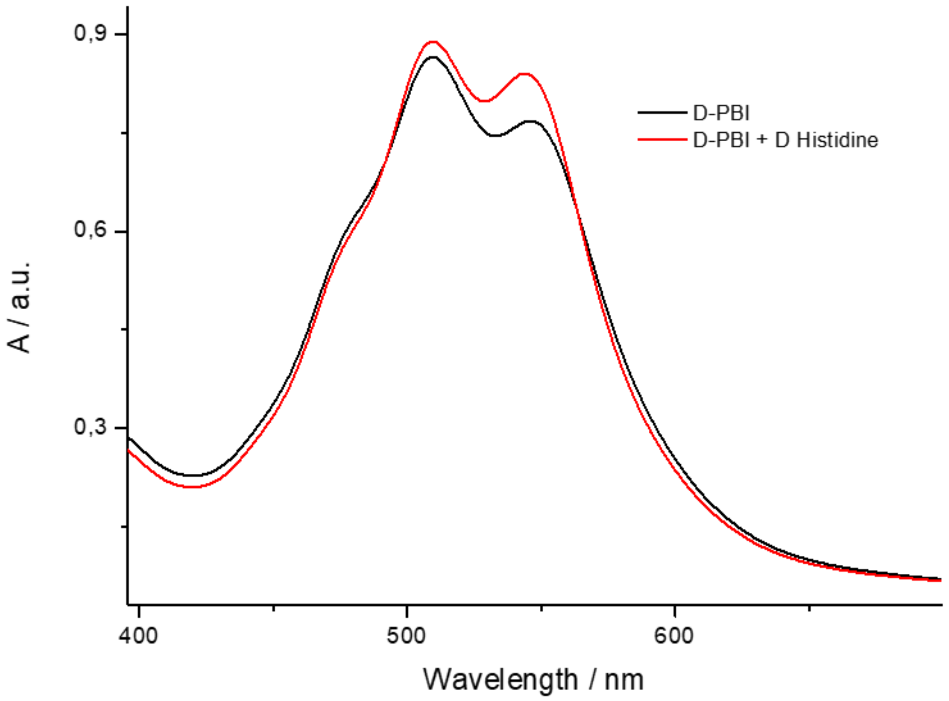

Figure S8: UV-visible spectra of D-PBI aqueous solutions (5x10^{-5} M) and mixed with D-histidine (10^{-4} M).
